# The effectiveness of alcohol label information for increasing knowledge and awareness: a rapid evidence review

**DOI:** 10.1186/s12889-023-16327-x

**Published:** 2023-07-31

**Authors:** Charlotte E. R. Edmunds, Natalie Gold, Robyn Burton, Maria Smolar, Matthew Walmsley, Clive Henn, Mark Egan, Anh Tran, Hugo Harper, Max Kroner Dale, Helen Brown, Kristina Londakova, Nick Sheron, Felix Greaves

**Affiliations:** 1 Health Improvement, Public Health England, Wellington House, 133-155 Waterloo Road, London, SE1 8UG UK; 2 Institute of Psychiatry, Psychology and Neuroscience, King’s College London, 16 De Crespigny Park, London, SE5 8AF UK; 3 Centre for Philosophy of Natural and Social Science, London School of Economics and Political Science, Houghton Street, London, WC2A 2AE UK; 4 Behavioural Practice, Kantar Public, 4 Millbank, Westminster, London, SW1P 3JA UK; 5 Behavioural Insights Team, 4 Matthew Parker St, Westminster, London, SW1H 9NP UK; 6 Institute of Hepatology, Foundation for Liver Research, 111 Coldharbour Lane, London, SE5 9NT UK; 7 School of Psychology, Bath Spa University, Bath, BA2 9BN UK; 8 Department of Primary Care and Public Health, Imperial College, London, UK South Kensington, London, SW7 2AZ UK

**Keywords:** Alcohol, Warning labels, Health literacy, Health risks, Risk communication

## Abstract

**Background:**

Consumers have difficulty understanding alcoholic units and low risk drinking guidelines (LRDG). Labelling may improve comprehension. The aims of this rapid evidence review were to establish the effectiveness of on-bottle labelling for (i) improving comprehension of health risks; (ii) improving comprehension of unit and/or standard drink information and/or LRDG, and (iii) reducing self-reported intentions to drink/actual drinking.

**Methods:**

Electronic database searches were carried out (January 2008-November 2018 inclusive). Papers were included if they were: published in English; from an Organization for Economic Co-operation and Development country; an experimental/quasi-experimental design. Papers were assessed for quality using the Effective Public Health Practice Project Quality Assessment tool. Ten papers were included. Most studies were moderate quality (n = 7).

**Results:**

Five themes emerged: comprehension of health risks; self-reported drinking intentions; comprehension of unit/standard drink information and/or LRDG; outcome expectancies; and label attention. Labelling can improve awareness, particularly of health harms, but is unlikely to change behaviour. Improved comprehension was greatest for labels with unit information and LRDG.

**Conclusions:**

Alcohol labelling can be effective in improving people’s comprehension of the health risks involved in drinking alcohol enabling them to make informed consumption decisions, and perhaps thereby provide a route to changing behaviour. Thus, effective alcohol labelling is an intervention that can be added to the broader suite of policy options. That being said, the literature reviewed here suggests that the specific format of the label matters, so careful consideration must be given to the design and placement of labels.

**Supplementary Information:**

The online version contains supplementary material available at 10.1186/s12889-023-16327-x.

## Introduction

Globally in 2017, among those aged 15–49 years, alcohol was the leading risk-factor for ill-health, disability and death, accounting for 6.5% of total disability adjusted life years (DALYs) [1]. Among all ages it was the seventh, accounting for 4.3% of DALYs. The burden of harm arises through alcohol’s place in society. Alcohol is a commonly consumed commodity, central to many cultural, religious and social practices. In 2016 global per capita consumption over 15 years was 6.4 L, with the highest levels seen in Europe at 9.8 L per capita, and 11.4 L in the UK [2].

Drinking guidelines vary internationally. UK guidelines are based around the concept of units (1 cl, 10ml, or 8 g of ethanol). There are around 2.5 units in a pint of beer, 2.3 units in a 175ml glass of wine and 1.4 units in a shot of spirits. The low risk drinking guideline (LRDG) is set at the level where 1% of deaths are alcohol-related and are communicated as follows: *“To keep health risks from alcohol to a low level, it is safest not to drink more than 14 units/week on a regular basis. If you regularly drink as much as 14 units/week, it is best to spread your drinking evenly over three or more days”* [3]. In England, 31% of men and 16% of women regularly consume more than 14 units/week [4], meaning they are at a greater risk of alcohol-related health and social harms [5]. Drinking within the LRDG helps minimise risks of harm [3], though it requires accurate monitoring by consumers [6]. Units are an important concept for conveying information about alcohol intake, though a major difficulty is the variability of % alcohol by volume (ABV) within and between beverage types across different sized measures [7]. Since drinkers cannot easily discriminate between % ABV in drinks [8], labelling or unit information at the point-of-purchase or point-of-consumption can facilitate monitoring. European Union (EU) regulations only mandate for the ABV to be displayed on alcoholic products though Member States are free to adopt national policies.^1^ The UK has a voluntarily agreement in place with drinks producers to display unit content, pregnancy warnings, and the LRDG, though adherence to these voluntary guidelines is poor [9].

Consumers have difficulty understanding units and LRDG. In England, one in four drinkers could report the LRDG, and even fewer reported using the guideline to monitor their own consumption [10]. Consumer understanding of alcohol harms is also low, in the UK, unprompted, only 40% of respondents identified liver damage/failure as a drinking outcome, and 31% reported cancer [11]. Despite the clear knowledge deficit, public support for alcohol labelling is high; 80% of a UK sample reported supporting or strongly supporting alcohol warning labels [12].

### Aims

The aims of this rapid evidence review were to establish the effectiveness of labelling approaches on bottles of alcohol for (i) improving comprehension of the health risks of consuming alcohol; (ii) improving comprehension of unit and/or standard drink (SD) information and/or a LRDG, and (iii) reducing self-reported intentions to drink/actual drinking.

The findings of this review were used to inform an experimental study which tested different label designs on an online sample of adult drinkers in England to understand their impact on consumer understanding of LRDGs [13].

## Methods

### Design

A rapid evidence review was used which balances resource and time constraints by streamlining the systematic review approach to synthesise evidence to inform decision makers [14]. An internal research protocol was developed by the Behavioural Insights Team with oversight from Public Health England, but not preregistered. Rapid reviews mirror the systematic review approach, but take ‘abbreviations’ or ‘accelerations’ to deliver a robust and transparent review to influence policy [15].

### Literature search

Electronic database searching included four databases (Medline, PsycInfo, Scopus, and Food Science and Technology Abstracts) and was carried out in November 2018. These databases were those the authors had access to that were relevant to the topic at hand. Papers published between January 2008-November 2018 (inclusive) were included. Database searching was supplemented by hand-searches on Google Scholar and consultations with expert groups listed in Supplementary Material 1. Searches were tailored for each database and based around the constructs of alcohol, labelling and outcomes of interest (full search terms in Supplementary Material 1).

### Eligibility criteria

To be eligible for inclusion in this review, papers needed to meet the following criteria:

- Published between January 2008-November 2018 (inclusive).
- Published in English.
- Data collected from Organization for Economic Co-operation and Development countries.
- Relevant outcome measures.
- Experimental/quasi-experimental.

Studies were excluded if the populations of focus were: under the legal drinking age; pregnant women; or those with alcohol dependence. While pregnant women are a key group in relation to alcohol health literacy, this review focuses on labelling messages for the general population and its findings will be used to inform an experimental study testing labels on the general population. Furthermore, pregnant women and children are advised to abstain from alcohol entirely and thus require different messaging to the general population. Studies evaluating the effectiveness of media campaigns/advertisements were excluded [13]. Editorials/duplicates/irretrievable/or studies with insufficient data were also excluded. Typically, studies with insufficient data were where the paper lacked meaningful details in an otherwise eligible paper. For instance, an author might have just reported a *p*-value for between groups differences without the mean score and error estimates. Research funded by the alcohol industry was also excluded in line with previous evidence reviews [9].

### Study selection

After applying the eligibility criteria, studies were further refined using a sequential process. Study titles and abstracts were scrutinised to determine eligibility. Following this, or when relevance was unclear from title and abstract alone, full texts were examined.

A test of inter-rater agreement was conducted by two researchers. Both conducted a title and abstract screen of 100 articles and found an agreement rate of 95%; this process produced a kappa statistic of 0.71, indicating a high level of agreement.

### Data extraction

Key variables were systematically extracted by one author using the PICO (population, intervention, comparator, outcomes(s)) framework [16]. Measures of association or effect and uncertainty estimates were extracted to be reported. Extraction templates used during the current study available from the corresponding author on reasonable request.

### Risk of bias

The Effective Public Health Practice Project (EPHPP) Quality Assessment tool was used to assess methodological quality [17]. This tool appraises potential selection bias, confounders, and issues of blinding, consistency and fidelity, and study design and analytical methods. Studies are rated as ‘strong’, ‘moderate’, or ‘weak’ quality. Quality appraisal was conducted by two independent reviewers with discrepancies resolved by local discussion.

## Results

### Study selection

**Fig. 1:**
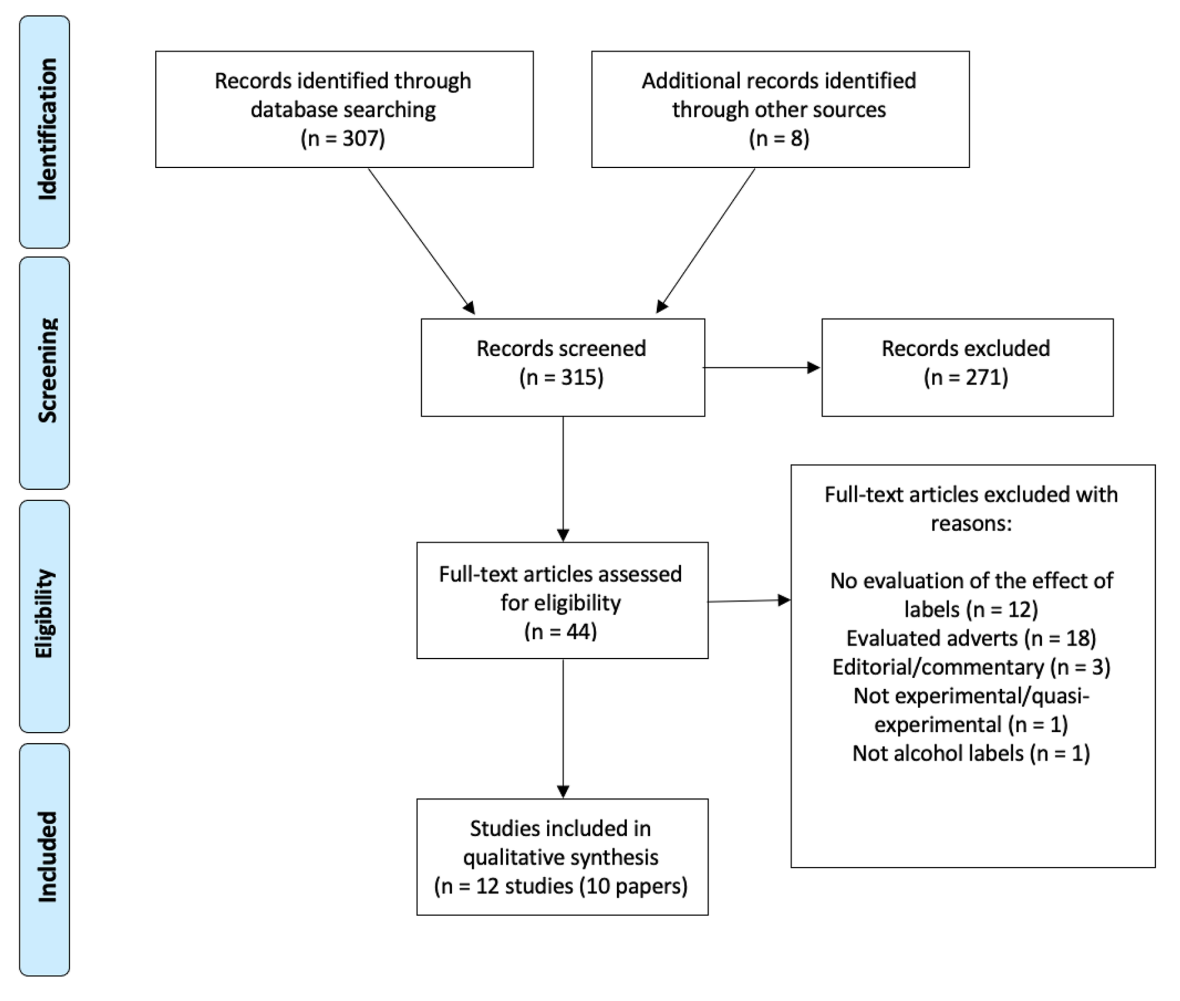
PRISMA flow diagram of studies identified in this review

The study selection process can be seen in Fig. 1, based on the Preferred Reporting Items for Systematic Reviews and Meta-Analyses (PRISMA) reporting guidelines [18]. In total 315 non-duplicate papers were initially identified. On the basis of title and abstract, 271 papers were excluded, leaving 44 to be evaluated based on the full-text. The majority were excluded at this stage because they did not use an experimental or quasi-experimental design or because they did not examine healthy (non-clinical population), non-pregnant adults. After screening 9 papers (10 separate studies) were included in the review.

Five studies were from Australia, three from the UK, two from Canada, one from the United States of America, and one which simultaneously recruited participants from Luxembourg and Germany. Most studies were moderate quality (n = 8), two weak, and two strong. Between-subjects designs, where participants were exposed to different experimental groups, were utilised in seven of the studies, with the remaining three utilising within-subjects designs, whereby the same participants were exposed to all conditions. An overview of study characteristics is given in Table 1.

**Table 1:** An overview of the studies included in the review

Reference (Country)	Study design	Study aim(s)	Participants *n*	Independent variable(s)	Dependent variable(s)	GRADE quality rating
(Blackwell, Drax, Attwood, Munafò, & Maynard, 2018) (UK [19])	Between-subjects design	To examine the impact of alcohol labels on (i) knowledge of weekly guidelines, and (ii) motivation to drink	1,884	Study 1) a) basic ABV label b) responsibility deal label c) food label equivalent d) pie chart Study 2) One of eight general or specific, positive or negatively framed warnings	Study 1) Accuracy of estimating weekly servings Study 2) Motivation to drink	Strong
(Jongenelis et al., 2018) (Australia [22])	Between-subjects design	To assess whether exposing drinkers to warning statements on alcohol products can increase their capacity to make healthier choices	364	One of five warnings: a) warning: alcohol increases your risk of cancer b) warning: alcohol increases your risk of diabetes c) warning: alcohol increases your risk of liver damage d) warning: alcohol increases your risk of mental illness e) warning: alcohol increases your risk of heart disease	The extent to which participants believed alcohol is a risk factor for: a) cancer b) diabetes c) liver damage d) mental illness e) heart disease	Moderate
(Pham, Rundle-Thiele, Parkinson, & Li, 2017) (Australia [28])	Study 1) within-subjects design Study 2) between-subjects design	To investigate the levels of attention paid to alcohol warning labels	Study 1) 559 Study 2) 87	Study 1) a) moderate size black written warning (control) b) red text instead of black (colour condition) c) original label size increased by 50% (size condition) d) red text and label size increased by 50% (colour and size condition) Study 2) a) healthy eating poster b) organ donation poster c) one of four randomly assigned wine warning labels (as in study 1)	Study 1) a) “How much attention did you pay to [labels]”^2^ b) “How much did you concentrate on [labels]”^3^ Study 2) a) Fixation count b) Fixation duration c) Time to first fixation	Study 1) Moderate Study 2) Moderate
(Hobin et al., 2017) (Canada [20])	Between-subjects design	To test the efficacy of alcohol labels with SD information and Canada’s LRDG compared to ABV% labels on consumers ability to estimate alcohol intake	2,016	Control label (current Canadian label regulations including ABV%) compared to one of five conditions: a) pictogram b) chart c) label listing number of SD per container d) SD information and LRDG as a pictogram e) SD information and LRDG as a chart	Participants were asked to estimate: a) the amount in a SD b) the number of SD in an alcohol container c) the number of SD to consume to reach the LRDG	Moderate
(Wigg & Stafford, 2016) (UK [23])	Between-subjects design	To test the effectiveness of a range of health warnings	60	a) no health warning (control) b) text only warning c) pictorial warning	a) level of fear arousal b) perceptions of health risk of consuming alcohol c) intentions to reduce and quit drinking	Low
(Miller, Ramsey, Baratiny, & Olver, 2016) (Australia [24])	Within-subjects design	To investigate the impact of cancer warning statements on consumer’s level of agreement, prompting conversation, influencing drinking behaviour, and educating others about cancer risk	1,547	a) three drinks a day increases your chance of cancer by 20% b) alcohol causes cancer c) two or more drinks a day can increase your risk of mouth and throat cancer by over 50% d) 1 in 5 breast cancers are caused by alcohol	Likert scale from “strongly agree” to “strongly disagree”: a) raise awareness about the link between regular alcohol consumption and cancer b) prompt conversations about the cancer risk involved in drinking alcohol regularly c) prompt me to drink alcohol less often d) prompt my friends to drink alcohol less often e) prompt me to talk to my family and/or friends about the cancer risk associated with alcohol f) prompt me to educate my children about the cancer risk associated with alcohol	Moderate
(Chen & Yang, 2015) (USA [25])	Within-subjects design	To examine whether risk perceptions of alcohol-attributable cancer influence alcohol consumption among students	127	a) text warning b) table warning c) graph warning	a) perceived susceptibility b) perceived severity	Moderate
(Krischler & Glock, 2015) (Luxemburg and Germany [27])	Between-subjects design	To investigate the effectiveness of tailored pictorial warning labels formulated as questions or statements.	122	a) Warning pictures expressed alongside questions b) Warning pictures expressed alongside statements c) Bottles with no warning statements	a) Depression-related outcome expectancies b) Socially-related outcome expectancies c) Tension-related outcome expectancies	Low
(Osiowy et al., 2015) (Canada [21])	Between-subjects design	To investigate whether standard drink labels would improve drinkers’ accuracy when estimating personal alcohol consumption.	301	2 × 3 × 3 experimental design: two label designs (%ABV, standard drink labels); three beverages (beer, wine and spirits); three beverage strengths (low, regular, high)	(a) relative and (b) absolute percent errors in their estimations of own drinking in comparison with correct answers	Strong
(Pettigrew et al., 2014) (Australia [26])	Between-subjects design	To investigate the acceptability of cancer warning statements for alcoholic beverages	2,168	Control health statement “Warning: alcohol harms your health” compared to three randomly shown statements from the following: a) Warning: alcohol increases your risk of cancer b) Alcohol causes cancer: reduce your intake to reduce your risk c) Reduce your drinking to reduce your risk of cancer d) Alcohol increases your risk of bowel cancer e) Alcohol increases your risk of breast cancer f) Alcohol increases your risk of breast, bowel, throat and mouth cancer g) Alcohol increases your risk of cancer h) Alcohol can cause breast cancer i) Alcohol can cause bowel cancer j) Alcohol causes around 5,000 new cases of cancer each year k) Alcohol causes 1 in 2 cancer deaths	Participants asked to report the extent to which they found the message: a) believable b) convincing c) personally relevant	Moderate

^2^ 7-point score from ‘none at all’ to ‘very much’.

^3^ 7-point score from ‘none at all’ to ‘very much’.

### Narrative synthesis

Five themes emerged and are presented as a narrative synthesis, as the papers were too diverse in measures and outcomes for any other approach. This involved looking for emerging themes inductively within the results and classifying them into groups. These were: comprehension of the health risks of alcohol; self-reported drinking intentions; comprehension of unit/SD information and/or LRDG; outcome expectancies; and label attention.

#### Comprehension of unit/standard drink information and/or low-risk drinking guidelines

Three between-subjects experiments tested the impact of labels on participants’ abilities to accurately identify units, SDs and the LRDG [Study 1 19–21]. In an online, between-subjects experiment, participants were randomly assigned to one of four unit label conditions: ABV%, the total units per bottle (which is used on many existing labels), food label equivalent, and a pie chart, and were assessed on the accuracy of estimating weekly servings [19]. So, for a beer the ABV% condition presented “ABV 4.8% 284ml” and the total units condition showed a picture of a bottle with “1.4 UK Units” printed inside. The food label equivalent presented a graphic that showed both the number of units and the percentage of the guideline weekly amount. Finally, the pie chart condition showed a pie chart where the number of wedges corresponded to the number of that drink one could have to meet the weekly LRDG of 14 units. One wedge was shaded and the number of units was printed inside. For all conditions, the average accuracy was below zero meaning, on average, participants underestimated the number of drinks they could consume within the UK weekly LRDG. This demonstrates the difficulty drinkers have estimating alcohol consumption. Compared to the ABV% and total units conditions, the food label and pie chart elicited better accuracy suggesting UK labelling can be improved to facilitate consumer comprehension.

A second between-subjects experiment aimed to understand the effectiveness of labels with SD information and Canada’s LRDG compared to only ABV% labels [20]. In an online survey, participants viewed an alcohol label and were asked to estimate the amount in a SD; the number of SDs in an alcohol container; and the number of SDs to consume to reach the recommended daily limit in Canada’s LRDG. They found labels with SD and LRDG information were more effective than ABV% labels for improving estimates of alcohol consumption. Participants assigned to SD information only or both SD and LRDG were more likely to correctly estimate the amount of alcohol in a SD and number of SDs in a container compared to those in the ABV% only. There was a 12.6–58.9% improvement in accuracy across all beverage types and outcomes when participants viewed labels with SD and LRDG information.

A third between-subjects experiment aimed to understand the effectiveness of labels with SD information compared to ABV% labels [21]. The experiment had a 2 × 3 × 3 experimental design: two label designs (ABV%, standard drink labels); three beverages; and three beverage strengths (low, regular, high). Each participant answered six questions for their preferred beverage (beer, wine or spirits). As a dependent variable, participants were asked how many SDs they would have drunk for a certain amount of their preferred beverage. They found that people were more accurate in the SD conditions than the ABV% conditions. They also found that participants in the beer conditions were more accurate than those in the other conditions. Additionally, participants were worse at judging for low strength beverages than high strength beverages. They also found that participants were less likely to underestimate the SD of beverages when given SD rather than ABV% labels.

These three studies suggest that not all labels are created equal: labels that help participants generalise to the weekly limit appear to help participants best understand drinking limit information. However, the latter two studies may have been influenced by their choice of dependent variables [20, 21]. Both papers rated participants’ understanding by asking them to judge in terms of SDs, without the corresponding test in terms of ABV%. Thus, the success of SDs might just be because it was an easier mathematical problem to solve.

#### Comprehension of alcohol-related health

Five studies explored the impact of labelling approaches on participants’ comprehension of alcohol’s health risks: 4 between-subjects designs and 1 within-subjects design [22–26].

An Australian between-subjects design aimed to assess whether exposure to alcohol warning statements relating to specific chronic diseases increases consumers’ beliefs that alcohol is a risk factor for those diseases [22]. Participants were drinking at levels associated with long-term risk of harm but were not considered to be dependent drinkers. For all conditions, except liver damage, the extent to which alcohol was believed to be a risk factor was greater after participants were exposed to a statement presenting information advising of such a risk. The effect sizes associated with these pre- to post-exposure changes were reasonable, especially for the diabetes (pre-test mean = 3.29, SD = 1.21; post-test mean = 4.35, SD = 0.70), mental illness (pre-test mean = 3.15, SD = 1.20; post-test mean = 4.07, SD = 0.83), and heart disease conditions (pre-test mean = 3.51, SD = 1.14; post-test mean = 4.34, SD = 0.87). The null finding relating to liver damage was likely due to the high baseline level of knowledge that alcohol causes liver damage (pre-test mean = 4.35, SD = 0.97; post-test mean = 4.50, SD = 0.89, highest possible rating = 5).

A between-subjects study aimed to investigate the effectiveness of alcohol labels on perceptions of the health risk of alcohol use [23]. Participants viewed one of three warnings: no health warning, a text-only warning, or a pictorial warning. Pictorial warnings were associated with increased perceptions of the health risks of consuming alcohol, though there was no difference between the control and text conditions.

A between-subjects study aimed to investigate the impact of cancer warnings on consumers’ level of agreement that alcohol causes cancer [24]. Overall, labels were well received with over 70% of participants agreeing that labels could raise awareness and prompt conversations about the cancer risk of alcohol.

An Australian between-subjects study aimed to investigate the acceptability of cancer warning statements for alcoholic beverages [26]. A control health statement (“Warning: alcohol harms your health”) was compared to two of 11 randomly shown statements that specifically reference the link between alcohol and cancer. Overall, they found that responses to cancer statements were neutral to favourable. The cancer messages were more impactful for participants that were younger, female and/or more highly educated. Positively framed messages, messages that talked about a specific cancer or those framed as “increasing risk” performed better than negatively framed messages, those referring to cancer in general, and those using the term ‘can cause cancer,’ respectively.

A within-subjects study recruiting students from the USA aimed to explore how message formats (text, table, graph) influence risk perceptions about alcohol-attributable cancer [25]. Results showed that textual messages were related to lower risk perceptions compared to both graphic and tabular messages, however there was no difference between the graphic and tabular conditions suggesting that they are equally effective for increasing risk perceptions.

These studies show that labels do help educate people about specific health risks, especially when current awareness of that specific disease/health complication is low. Further, two suggests that warning labels of this kind would be well received by consumers [24, 26].

#### Self-reported drinking intentions

In an online, between-subjects experiment, participants were randomised to view one of eight health warnings that varied in their specificity (e.g. “cancer” vs. “bowel cancer”), framing (e.g. positive “drinking less reduces your risk” vs. negative “alcohol increases your risk”) and health message (e.g. “cancer” vs. “mental illness”) [Study 2 19]. Specificity did not appear to affect self-reported drinking intentions. The negatively framed warnings received higher scores for motivation to drink less than the positively framed warnings. Motivation to drink less was higher for those that received cancer messages than those that received mental health warnings.

An Australian between-subjects design aimed to assess whether exposure to warning statements relating to specific chronic diseases influences consumption intentions [22]. Participants were drinking at levels associated with long-term risk of harm but not considered to be dependent. A decrease in self-reported intentions to drink was observed for participants exposed to the cancer, diabetes, and mental illness statements, but not for heart disease or liver damage (where intention was a composite score, which averaged three questions asking about the extent to which: (i) they believed they should reduce the amount of alcohol they consume, (ii) they expected that they will actually reduce the amount of alcohol they consume and (iii) they intended to consume five or more drinks in a single sitting within the following two weeks). This may be partially attributed to the relative novelty of the former as reflected in lower baseline belief levels.

A between-subjects design recruiting participants from Luxemburg and Germany aimed to investigate the effectiveness of tailored pictorial warning labels formulated as questions, e.g. “Do you really want alcohol to help you test your limits?” or statements, e.g. Yes, alcohol helps test your limits” [27]. No significant impact was observed for self-reported drinking intentions.

In a between-participants study, participants viewed one of three warnings: no warning, text-only warning, or a pictorial warning [23]. Pictorial warnings were associated with increased intentions to reduce and quit drinking. Text warnings were no different from control.

These studies suggest that vivid labels (either because they describe concrete, frightening diseases or because they are images) are best at reducing participants’ intentions to drink. Formulating the labels as questions does not appear to affect self-reported intentions to drink less.

#### Outcome expectancies

A single between-subjects design explored the impact of message framing on alcohol outcome expectancies – a measure of the perceived expectancies associated with consuming alcohol (can be positive or negative) [27]. Participants were recruited from Luxemburg and Germany and pictorial warning labels were presented with questions or statements [27]. For positive outcome expectancies there were no differences for any of the conditions. For negative expectancies, there were no differences between participants in the statements or control group, whereas participants in the question group reported increased levels of negative alcohol outcome expectancies relative to controls. This suggests that question framing facilitates understanding of the negative outcomes of alcohol.

#### Label attention

A multi-method within-subjects design was used to deliver four labelling conditions: (i) a control, (ii) enhanced colour, (iii) size, and (iv) enhanced colour *and* size [28]. The first study used self-report survey to measure attention (n = 559) and the second used eye-movements (a more objective measurement) (n = 87). Average self-reported attention scores increased slightly from the control (i) and colour (ii) conditions (mean = 5.0, SD = 1.3 and mean = 5.1, SD = 1.2 respectively), to the size (iii) condition (mean = 5.2, SD = 1.2), with the highest scores for the colour and size (iv) condition (mean = 5.4, SD = 1.2). Objective measures using eye-movements showed attention levels were low; only 65.5% of the sample looked at the labels. A higher proportion of the sample looked at the label when changes in colour and size were made (81%). There were no differences in terms of number of fixations, time to first fixation or fixation duration between groups, which perhaps suggests that labels may need to be far more prominent to attract attention.

## Discussion

The aims of this rapid review were to establish the effectiveness of approaches to alcohol labelling for improving comprehension of units, the LRDG, and alcohol-related health risks, and any effect of labelling on self-reported intentions to drink/actual drinking. The review identified 11 studies (ten experiments).

A range of labelling approaches were effective at increasing participant comprehension, particularly for approaches that used pictorial warnings [23] and messages relating to cancer [22, 24]. These conclusions are based on a small number of moderate/weak studies, with inconsistencies in the strength, and sometimes direction, of findings.

The effect of labels on drinking intentions were mixed, and no studies reported on actual drinking. A single strong study demonstrated motivations to drink less were higher for cancer and negatively framed messages [19], but these motivations may not translate into changes in drinking. Though pictorial warnings tended to result in greater intentions to reduce drinking [23], this finding was not consistent across studies [27]. A single study reported higher intentions to reduce drinking for health warnings limited to some messages (e.g. cancer), and not to others (e.g. liver disease – likely due to a high baseline knowledge) [22]. The weight of evidence is unconvincing for the effectiveness of labels for reducing drinking intentions. This aligns with previous findings that explored the impact of campaigns and adverts and showed an improvement in awareness but not behaviour [9]. Nonetheless, indications that a product is hazardous is a fundamental consumer right, and consumer awareness can increase support for more stringent alcohol policies, such as taxation [29]. Labelling is an important component of any overall policy approach as per best practice recommendations in the sphere of alcohol policy [9, 30, 31].

This review highlighted a lack of awareness of unit (or equivalent) information and LRDG. It suggests that enhanced labels can reduce this knowledge deficit, particularly when both unit and LRDG information is given simultaneously [19, 20]. It is possible that these components of labelling simultaneously enable a better understanding suggesting that UK labelling initiatives can be improved.

A lack of clear evidence precludes firm conclusions relating to message framing and outcome expectancies [27]. Further research would improve understanding. The dissonance between subjective and objective measures of label attention [28] could also be resolved by future research using objective measures. Though label attention is low, this could be due to their size or prominence. Any implementation needs to consider not just content, but also size, font, and placing. Important lessons could be learned from the experience of tobacco and food - tobacco labelling lowered initiation rates and increased cessation rates [32, 33], and nutrition labels increased consumer choices of healthier alternatives [34].

This review informed an experimental study testing different label designs on an online sample of adult drinkers in England to understand their effect on consumer understanding that has since been published [13]. Given our findings that there is a lack of awareness of SDs and the LRGDs, the experiment tested whether different label designs could improve knowledge of the UK’s LRDG, which recommend not regularly drinking more than 14 units of alcohol per week, and improve their understanding of how many servings and containers of alcoholic drinks would take them to 14 units. The study found that labels with enhanced pictorial representations of alcohol content improved understanding compared with industry-standard labels. Indeed, the designs that resulted in the best understanding included the low-risk drinking guidelines in a separate statement located beneath the graphics.

This review, and the subsequent experiment, supports more recent work that investigated alcohol labels in Yukon, Canada [35–39]. They found that strengthening health messages (including a health warning, standard drink, or national guidelines) on alcohol containers increased consumer attention to and processing of labels [35, 36], as well as increasing consumers’ knowledge of the health implications of alcohol [37]. It also reduced consumers’ self-report of alcohol consumption [35], intentions to drink [36] and actual alcohol purchasing [39].

### Limitations

The studies in this review typically measured a single (or small number of) label exposures at a solitary time-point in contrast to repeated exposures experienced in day-to-day life. It is possible that responses, such as changes in drinking, only develop after repeated exposures to information received on alcohol labels or in tandem with other skills building and behaviour change interventions. Indeed, the research reviewed here suggests that warning labels are typically low intensity – small and difficult to notice [28]. Alternatively, the initial impact of labels may diminish with time, akin to ‘familiarity breeds contempt’. Further, it suggests that emotive/impactful warnings yield better outcomes than their less impactful counterparts [22, 23]; however, emotional impact may decrease with exposure. To maintain label impact, it may be necessary to continuously intensify both the exposure and intensity of the labels (as has been the case for tobacco) [40]. Such a strategy has limits and one alternative might be to *periodically* introduce high-intensity labels to recapture the initial impact. That being said, previous research that focused on the familiarity of the item, rather than familiarity with the warning label, found that familiarity tended to improve compliance with the label [41]. This suggests future work exploring the interaction between intensity and familiarity of warnings is likely to be important.

Finally, the use of a rapid review protocol leaves this work more vulnerable to bias and errors. For instance, the search process in rapid reviews is typically less comprehensive. To try and compensate for this, we consulted expert groups for potentially missing literature (see Supplementary Material 1). Additionally, this approach is best suited to answering a focused question. Here, this resulted in us focusing on the results from experimental or quasi-experimental studies. Although these studies are easier to interpret, there are also not as many of them in this field which limits our findings somewhat. Further, these studies expose an online panel sample to the labels, whereas exposure to on-bottle labels in a field study would yield a more representative sample of alcohol purchasers, as well as potentially different responses by participants.

## Conclusions

Well-implemented alcohol labels play an important role in increasing comprehension of risk and understanding of units and LRDGs, although there is room for improvement in label design. This understanding underpins a fundamental right to knowledge and can enable informed choice. Public support for labelling is high [24]. Opportunities exist to improve label design. Effective labelling is thus an intervention that can be added to the broader suite of policy options [9].

## Electronic supplementary material

Below is the link to the electronic supplementary material.

Supplementary Material 1

## Data Availability

All relevant data is provided in Table 1. All data generated or analysed during this study are included in this published article.

## Footnotes

1 Note that although the UK has now left the EU, EU legislation on alcohol labels remains until replaced by new UK legislation.
