# Correction: The effectiveness of alcohol label information for increasing knowledge and awareness: a rapid evidence review

**DOI:** 10.1186/s12889-025-21971-6

**Published:** 2025-03-14

**Authors:** Charlotte E. R. Edmunds, Natalie Gold, Robyn Burton, Maria Smolar, Matthew Walmsley, Clive Henn, Mark Egan, Anh Tran, Hugo Harper, Max Kroner Dale, Helen Brown, Kristina Londakova, Nick Sheron, Felix Greaves

**Affiliations:** 1 Health Improvement, Public Health England, Wellington House, 133-155 Waterloo Road, London, SE1 8UG UK; 2 Institute of Psychiatry, Psychology and Neuroscience, King’s College London, 16 De Crespigny Park, London, SE5 8AF UK; 3 Centre for Philosophy of Natural and Social Science, London School of Economics and Political Science, Houghton Street, London, WC2A 2AE UK; 4 Behavioural Practice, Kantar Public, 4 Millbank, Westminster, London, SW1P 3JA UK; 5 Behavioural Insights Team, 4 Matthew Parker St, Westminster, London, SW1H 9NP UK; 6 Institute of Hepatology, Foundation for Liver Research, 111 Coldharbour Lane, London, SE5 9NT UK; 7 School of Psychology, Bath Spa University, Bath, BA2 9BN UK; 8 Department of Primary Care and Public Health, Imperial College, London, UK South Kensington, London, SW7 2AZ UK

**BMC Public Health (2023) 23:1458**

10.1186/s12889-023-16327-x

The original publication of this article contained an incorrect competing interests statement. The incorrect and correct information is listed in this correction article. The original article has been updated.

Incorrect competing interests

There are no competing interests.

Correct competing interests

The Authors, Mark Egan, Hugo Harper, Max Kroner Dale, Helen Brown and Kristina Londakova worked for the Behavioural Insights Team (BIT) that has done research for, and/or collaborated with the alcohol industry, in the form of the Drinkaware Trust.

