# Supplementary material for: The effectiveness of alcohol label information for increasing knowledge and awareness: a rapid evidence review
Source: BMC Public Health. 2023 Jul 31;23:1458. doi: 10.1186/s12889-023-16327-x (PMC10388453; doi:10.1186/s12889-023-16327-x)
Supplement: Supplementary file 1 — Supplementary Material 1 [file 12889_2023_16327_MOESM1_ESM.docx]

**9. Appendices**

**9.1 Appendix 1 – expert groups consulted for potentially missing literature**

The Behaviour and Health Research Unit at the University of Cambridge; the Winton Centre for Risk & Evidence Communication at the University of Cambridge; (iii) the Tobacco and Alcohol Research Group at Bristol University; (iv) the Drug and Alcohol Research Centre at Middlesex University; (v) Professor Nick Sheron at the Institute of Hepatology, King’s College London; and (vi) Professor Betsy Thom at Middlesex University.

**9.2 Appendix 2 – search strategies**

**Medline**

Database(s): Ovid MEDLINE(R) and Epub Ahead of Print, In-Process & Other Non-Indexed Citations, Daily and Versions(R) 1946 to December 14, 2018

1 exp *Alcohol Drinking/pc [Prevention & Control]

2 (problem* adj2 drink*).tw.

3 (alcohol-related adj (harm* or problem*)).tw.

4 (alcohol adj (abuse or misuse or consumption)).tw.

5 ((heavy or dependent or excessive or hazardo?s or harmful or $regular* or "too much") adj (drink* or alcohol*)).tw.

6 or/1-5

7 ((risk* or harm*) adj1 (perception* or perceive* or understand* or know* or belief* or believe or aware* or attitude*)).tw.

8 *Health Literacy/mt [Methods]

9 (health adj (literacy or knowledge)).tw.

10 7 or 8 or 9

11 *Health Promotion/mt [Methods]

12 (message* or promotion* or education* or campaign* or program* or intervention* or strateg* or label* or warning*).tw.

13 11 or 12

14 6 and 10 and 13

15 limit 14 to (english language and humans)

**PsycInfo**

Database(s): PsycINFO 2002 to December Week 2 2018

1 exp *alcohol abuse/

2 (problem* adj2 drink*).tw.

3 (alcohol-related adj (harm* or problem*)).tw.

4 (alcohol adj (abuse or misuse or consumption)).tw.

5 ((heavy or dependent or excessive or hazardo?s or harmful or $regular* or "too much") adj (drink* or alcohol*)).tw.

6 or/1-5

7 *risk perception/

8 ((risk* or harm*) adj1 (perception* or perceive* or understand* or know* or belief* or believe or aware* or attitude*)).tw.

9 *health literacy/ or *health knowledge/

10 (health adj (literacy or knowledge)).tw.

11 or/7-10

12 *health promotion/

13 (message* or promotion* or education* or campaign* or program* or intervention* strateg* or label* or warning*).tw.

14 12 or 13

15 6 and 11 and 14

**Scopus**

((TITLE-ABS ((heavy OR dependent OR excessive OR hazardo?s OR harmful OR regular* OR "too much") W/0 (drink* OR alcohol*))) OR (TITLE-ABS (alcohol-related W/0 (harm* OR problem*))) OR (TITLE-ABS (problem* W/2 drink*))) AND ((TITLE-ABS ((risk* OR harm*) W/1 (perception* OR perceive* OR understand* OR know* OR belief* OR believe OR aware* OR attitude*))) OR (TITLE-ABS (health W/0 (literacy OR knowledge)))) AND ((TITLE-ABS ("health promotion")) OR (TITLE-ABS (message* OR promotion* OR education* OR campaign* OR program* OR intervention* OR strateg* OR label* OR warning*)))

**Food Science and Technology Abstracts**

S1 DE "ALCOHOLIC BEVERAGES"

S2 DE "CONSUMER RESPONSE" OR DE "ACCEPTABILITY" OR DE "ACCEPTANCE" OR DE "CONSUMER ATTITUDES" OR DE "CONSUMER AWARENESS" OR DE "CONSUMER BEHAVIOUR" OR DE "CONSUMER CHOICE" OR DE "CONSUMER COMPLAINTS" OR DE "CONSUMER CONCERNS" OR DE "CONSUMER EXPECTATIONS" OR DE "CONSUMER LIKING" OR DE "CONSUMER OPINIONS" OR DE "CONSUMER PERCEPTION" OR DE "CONSUMER SATISFACTION" OR DE "DEMAND" OR DE "PARENTAL RESPONSE" OR DE "PREFERENCE"

S3 DE "DRINKING HABITS"

S4 S2 OR S3

S5 DE "LABELLING"

S6 DE "MARKETING"

S7 DE "ADVERTISING"

S8 S5 OR S6 OR S7

S9 S1 AND S4 AND S8
